# Genome Assembly and Annotation of the Medicinal Plant *Calotropis gigantea*, a Producer of Anticancer and Antimalarial Cardenolides

**DOI:** 10.1534/g3.117.300331

**Published:** 2017-12-12

**Authors:** Genevieve M. Hoopes, John P. Hamilton, Jeongwoon Kim, Dongyan Zhao, Krystle Wiegert-Rininger, Emily Crisovan, C. Robin Buell

**Affiliations:** Department of Plant Biology, Michigan State University, East Lansing, Michigan 48824

**Keywords:** *Calotropis gigantea*, cardenolide, Apocynaceae family, genome assembly, pharmaceutical, Genome Report

## Abstract

*Calotropis gigantea* produces specialized secondary metabolites known as cardenolides, which have anticancer and antimalarial properties. Although transcriptomic studies have been conducted in other cardenolide-producing species, no nuclear genome assembly for an Asterid cardenolide-producing species has been reported to date. A high-quality *de novo* assembly was generated for *C. gigantea*, representing 157,284,427 bp with an N50 scaffold size of 805,959 bp, for which quality assessments indicated a near complete representation of the genic space. Transcriptome data in the form of RNA-sequencing libraries from a developmental tissue series was generated to aid the annotation and construction of a gene expression atlas. Using an *ab initio* and evidence-driven gene annotation pipeline, 18,197 high-confidence genes were annotated. Homologous and syntenic relationships between *C. gigantea* and other species within the Apocynaceae family confirmed previously identified evolutionary relationships, and suggest the emergence or loss of the specialized cardenolide metabolites after the divergence of the Apocynaceae subfamilies. The *C. gigantea* genome assembly, annotation, and RNA-sequencing data provide a novel resource to study the cardenolide biosynthesis pathway, especially for understanding the evolutionary origin of cardenolides and the engineering of cardenolide production in heterologous organisms for existing and novel pharmaceutical applications.

Species within the Apocynaceae family have traditionally been used to treat fever, pain, and malaria in the Asia-Pacific region, primarily due to their production of secondary metabolites with antiplasmodial properties (Chan *et al.* 2016). Some of these secondary metabolites have also been found to contain antiproliferative properties and have been used to treat cancer. *Calotropis gigantea* (2*n* = 2*x* = 22) (Raghavan 1957) (Figure 1), a member of the Apocynaceae subfamily Asclepiadoideae, is known to produce several cardenolides or cardiac glycosides including calactin, calotoxin, calotropin, frugoside, and gofruside (Chan *et al.* 2016). Cardenolides are C_23_ steroids with a butenolide ring at C-17, and are synthesized in plants from mevalonic acid via phytosterol and pregnane intermediates (Bauer *et al.* 2010; Pandey *et al.* 2016) (Figure 1D). While cardenolides have previously been used as clinical drugs for congestive heart failure, they have recently been found to selectively inhibit cancer cells through the induction of apoptosis via complex cell signal transduction pathways associated with the Na^+^/K^+^ ATPase (Mijatovic and Kiss 2013).

**Figure 1:**
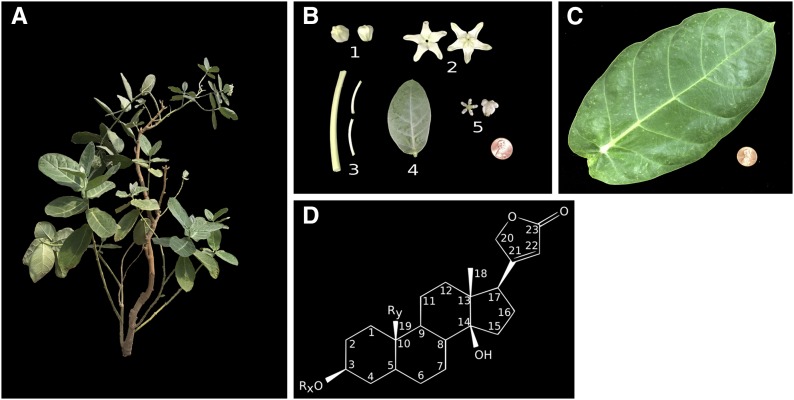
*C. gigantea* and cardenolide metabolites. (A) *C. gigantea* plant. (B) Plant tissues used for RNA-sequencing libraries. 1, closed bud 2; sepals and petals; 3, peduncle and pedicel; 4, young leaf; and 5, gynostegium. (C) Mature leaf tissue used for RNA-sequencing libraries. (D) Cardenolide metabolite with the carbons numbered. R_x_ and R_y_ indicate ambiguity of the attached groups, which vary depending on the specific cardenolide.

Cardenolides are currently harvested from plant tissue, a limited resource, and it is crucial to determine the complete biosynthetic pathway to engineer the production of cardenolides in heterologous organisms for pharmaceutical use. While cardenolide-producing species are distributed across 12 angiosperm families, they predominantly occur in the Asterid clade (Agrawal *et al.* 2012), including the Apocynaceae family, in which published genome sequences are available for two noncardenolide-producing species, *Catharanthus roseus* (Kellner *et al.* 2015) and *Rhayza stricta* (Sabir *et al.* 2016). Access to an annotated genome for an Asterid cardenolide-producing species would facilitate discovery of the genes involved in cardenolide biosynthesis through comparative genomics, coexpression analyses, and data mining of physically clustered genes, a feature of some plant specialized metabolic pathways (Nützmann *et al.* 2016). Here, we present an annotated draft genome sequence for *C. gigantea* and demonstrate the utility of this sequence in elucidating evolutionary relationships in the Apocynaceae family.

## Materials and Methods

### Genome assembly and analyses

Genomic DNA was extracted from young leaf tissue via the CTAB method (Saghai-Maroof *et al.* 1984), and used to construct three Illumina-compatible (Illumina, San Diego, CA) paired-end libraries with estimated insert sizes of 168, 291, and 519 bp (Supplemental Material, Table S1 in File S1), as previously described (Hardigan *et al.* 2016). Four Nextera Mate Pair libraries with estimated inner distances ranging from 2.7 to 8.8 kb were constructed per the manufacturer’s instructions (Table S1 in File S1). The libraries were sequenced on an Illumina HiSeq 2500 to generate paired-end 150 nt length reads. Read quality was assessed with FastQC (v0.11.5) (Andrews 2010), and adapters and bases of low quality (*Q* < 20) were removed with Cutadapt (v1.11) (Martin 2011), retaining reads >81 nt in length. Mate pair library reads were further processed with NextClip (v1.3.1) (Leggett *et al.* 2014), retaining paired reads >36 nt in groups A, B, and C (Table S1 in File S1). K-mers (17, 23, 33, 43, and 63) were counted using JellyFish2 (v2.2.6) (Marçais and Kingsford 2011) with ALLPATHS-LG (v52488) (Gnerre *et al.* 2011) error-corrected reads from all fragment libraries.

Two common assemblers capable of handling heterozygous genomes, ALLPATHS-LG (Gnerre *et al.* 2011) and Platanus (Kajitani *et al.* 2014), were used to generate *de novo* genome assemblies. As ALLPATHS-LG has a fragment library size restriction (less than two times the insert size), only cleaned reads from the 168 bp paired-end library (numbering 204,292,990) and all processed mate pair library reads (34,232,248) were used with the “haploidify” option in ALLPATHS-LG (v52488) (Gnerre *et al.* 2011), while cleaned reads from all libraries (278,764,758) and all mate pair library reads were used with the default options in Platanus (v1.2.4) (Kajitani *et al.* 2014). After filtering both assemblies for scaffolds >1000 bp, BUSCO (v2.0) (Simão *et al.* 2015) was run with the Embryophyta database on both assemblies to determine the representation of conserved plant orthologs.

To reduce the number and size of the gaps, GapCloser (v1.12r6) from SOAPdenovo (Luo *et al.* 2012) was run with the 291 and 519 bp fragment library paired-end reads (148,943,536), with maximum read length of 150 bp and the overlap parameter set to 31. To identify any contaminants in the assembly, BLASTN (BLAST+ v2.2.31) (Camacho *et al.* 2009) was used to search the gap-closed genomic assembly against the National Center for Biotechnology Information (NCBI) nr database (NCBI Resource Coordinators 2016). BUSCO (v2.0) (Simão *et al.* 2015) was subsequently run with the Embryophyta database on the final genome assembly.

BWA-MEM (v0.7.15) (Li and Durbin 2009) was used to align the cleaned reads from all fragment libraries to the assembly, marking secondary alignments (-M). Picard MarkDuplicates (v2.1.1; http://broadinstitute.github.io/picard) was used to mark PCR and optical duplicates. Picard-processed alignments were realigned around InDels using the GATK RealignerTargetCreator and IndelRealigner tools (v3.7.0; McKenna *et al.* 2010) with default options. The HaplotypeCaller tool was subsequently used to call variants with the standard_min_confidence_threshold_for_calling, min_base_quality, heterozygosity, and indel_heterozygosity options set to 30, 20, 0.05, and 0.001, respectively (v3.7.0; McKenna *et al.* 2010). Variants passed filtering with the GATK VariantFitration tool if they had read depth between 50 and 200, a quality depth >7, and a BaseQRankSum value between −5 and 5 (v3.7.0; McKenna *et al.* 2010). Heterozygous variants were further filtered such that only variants with a 30% ≤ allele frequency ≥ 70% were included in the analyses.

### Transcriptome assemblies and analyses

RNA was extracted from closed bud, sepal and petal, young leaf, peduncle and pedicel, gynostegium, and mature leaf tissues (Figure 1, B and C) via the QIAGEN RNeasy Plant Mini Kit, according to the manufacturer’s instructions (QIAGEN, Hilden, Germany), and treated with Turbo DNase to remove any DNA from the samples. Six KAPA Biosystems (Wilmington, MA) stranded libraries with NEBNext indices (Ipswich, MA) were constructed for each of the tissues and sequenced on an Illumina HiSeq 2500 platform, generating 50 nt single-end reads (Table S2 in File S1). The RNA-seq reads were then processed with FastQC (v0.11.5; Andrews 2010) and Cutadapt (v1.11; Martin 2011) as described above, retaining reads >30 nt in length.

*De novo*-assembled transcriptomes were generated for each RNA-seq library using Trinity (v2.3.2; Grabherr *et al.* 2011) in the strand-specific mode, and transcripts shorter than 500 bp were removed from the assemblies. The transcripts were aligned to the genome assembly using GMAP (v20161107; Wu and Watanabe 2005) with a 95% coverage and identity cutoff, and unaligned transcripts were searched against the nr database (NCBI Resource Coordinators 2016) using BLASTN from BLAST+ (v2.2.31; Camacho *et al.* 2009) with an *E*-value cutoff of 1e^−5^. Cleaned reads for each library were also aligned to the genome assembly using TopHat2 (v2.1.1; Kim *et al.* 2013) and Bowtie2 (v2.2.9; Langmead and Salzberg 2012) in stranded mode, with a maximum intron length of 30 kb.

### Genome annotation

A *C. gigantea*-specific custom repeat library (CRL) was created with RepeatModeler (v1.0.8; http://repeatmasker.org) using scaffolds >10 kb as input. The CRL was searched against a curated library of plant protein-coding genes and sequences, removing matches with ProtExcluder (v1.1; Campbell *et al.* 2014), and then combined with the RepBase (v20150807; Jurka 1998) Viridiplantae repeats to create a final CRL. The genome assembly was then masked with RepeatMasker (v4.0.6; http://repeatmasker.org) using the CRL with the –s option.

Genome-guided transcript assemblies were constructed for each RNA-seq library using Trinity (v2.3.2; Grabherr *et al.* 2011) and transcripts shorter than 500 bp were removed from the assemblies. *Ab initio* gene prediction was performed by training Augustus (v3.2.2; Stanke *et al.* 2006), using the hints provided by the alignments of the mature leaf RNA-seq library and the soft-masked genome assembly. Gene predictions were then obtained by running Augustus (v3.2.2) on the hard-masked genome assembly and refined using PASA2 (v2.0.2; Haas *et al.* 2003), utilizing the genome-guided transcript assemblies as evidence. To identify high-confidence gene models, the working set of gene models was searched against PFAM (v29; Finn *et al.* 2016) using HMMER (v3.1b2; Mistry *et al.* 2013). Expression abundances were also calculated using Cufflinks2 (v2.21; Trapnell *et al.* 2010) with the RNA-seq alignments described above. High-confidence gene models are defined as containing a PFAM hit and/or expression evidence in at least one RNA-seq library. Functional annotation was generated by searching the gene models against the *Arabidopsis thaliana* proteome (TAIR10; Lamesch *et al.* 2012), Swiss-Prot (Bairoch and Apweiler 2000), and PFAM (v29; Finn *et al.* 2016), and assigning a function in the same order.

### Comparative genomics analyses

Orthologous and paralogous genes in the three Apocynaceae species, along with the annotated proteomes of *Amborella trichopoda* (Amborella Genome Project 2013) and *A. thaliana* (Lamesch *et al.* 2012; Table S3 in File S1), were determined using OrthoFinder (v1.1.4; Emms and Kelly 2015) using default settings. To evaluate synteny between the species in the Apocynaceae family, MCScanX (Wang *et al.* 2012) was used with *R. stricta*, *C. roseus*, and *C. gigantea*. The predicted proteomes for the three species were searched against each other using BLASTP from BLAST+ (v2.5.0; Camacho *et al.* 2009), with an *E*-value cutoff of 1e^−5^ and a maximum of five hits reported.

### Identification of putative cardenolide biosynthetic enzymes

The *C. gigantea* proteome was searched against functionally characterized 3β-hydroxysteroid dehydrogenases (3βHSD; Herl *et al.* 2007) and progesterone 5β-reductases (P5βR; Bauer *et al.* 2010) using BLASTP from BLAST+ (v2.5.0; Camacho *et al.* 2009) with an *E*-value cutoff of 1e^−5^. Matches with percent identity >50% and query coverage >85% were retained. The identified *C. gigantea* proteins were aligned with the characterized 3βHSD and P5βR proteins using MUSCLE (Edgar 2004) as implemented in MEGA7 (Kumar *et al.* 2016). Neighbor-joining gene trees were constructed using default parameters with pairwise deletion and 1000 bootstrap replicates in MEGA7 (Kumar *et al.* 2016). Previously obtained fragments per kilobase exon model per million mapped reads (FPKM) values from Cufflinks2 (v2.21; Trapnell *et al.* 2010) for the identified genes were log2-transformed and hierarchically clustered in R (v3.4.2) using the “gplots” package (https://CRAN.R-project.org/package=gplots) “heatmap.2” function.

### Data availability

Raw sequence reads have been deposited with the NCBI under BioProject PRJNA400797. The genome assembly, annotation, and expression matrix have been deposited in the Dryad Digital Repository (DOI: 10.5061/dryad.fk41r) and the Medicinal Plant Genomics Resource (http://medicinalplantgenomics.msu.edu/). The supplementary tables are included in File S1.

## Results and Discussion

### De novo genome assembly generation

**Figure 2:**
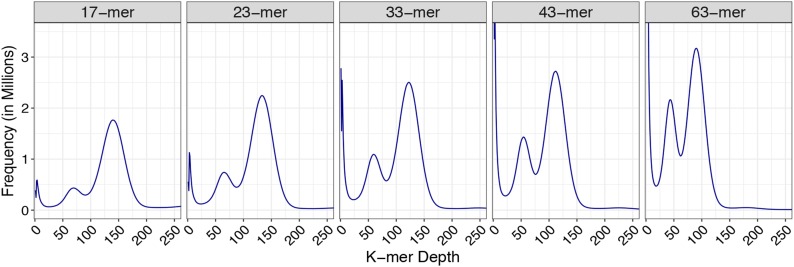
Heterozygosity of the *C. gigantea* genome and assembly. K-mer frequency plots generated in Jellyfish2 (Marçais and Kingsford 2011) using ALLPATHS-LG (Gnerre *et al.* 2011) error-corrected genomic fragment reads.

Flow cytometry of *C. gigantea* leaf tissue revealed a genome size of 225 Mb, and the genome was sequenced on an Illumina HiSeq platform to 193× average genomic coverage with the fragment library reads. To determine the relative level of heterozygosity in the genome, five k-mer lengths (17, 23, 33, 43, and 63) were counted (Figure 2). In the 17-mer plot, there are two peaks at k-mer depths of 140 and 69 (∼2:1 ratio), corresponding to homozygous and heterozygous regions in the genome, respectively. The ratio between the homozygous peak:heterozygous peak is 4:1 and 1.5:1 in the 23-mer plot and 63-mer plot, respectively. The presence of the small heterozygous peak and the disproportionate increase in the heterozygous peak relative to the homozygous peak as the k-mer size increases is indicative of a slightly heterozygous genome.

Two common assemblers capable of handling heterozygous genomes, ALLPATHS-LG (Gnerre *et al.* 2011) and Platanus (Kajitani *et al.* 2014), were used to generate *de novo* genome assemblies. Both ALLPATHS-LG, via the “haploidify” option, and Platanus contain algorithms that select a single haplotype from heterozygous regions to be incorporated into the assembly, resulting in a mixture of the two haplotypes in the final assembly (Gnerre *et al.* 2011; Kajitani *et al.* 2014). ALLPATHS-LG generated an assembly of 157,408,176 bp with an N50 scaffold size of 806,518 bp and Platanus generated an assembly of 146,937,509 bp with an N50 scaffold size of 187,271 bp (Table 1). The ALLPATHS-LG and Platanus assemblies contained 91.3% (87.8% complete and 3.5% fragmented) and 85.1% (77.2% complete and 7.9% fragmented) of BUSCO Embryophyta orthologs (Table 1), respectively. As ALLPATHS-LG generated a less fragmented, more complete assembly representing more of the genic space, subsequent work was performed with the ALLPATHS-LG assembly.

**Table 1:** Genome assembly metrics

		ALLPATHS-LG Assembly	PLATANUS Assembly	ALLPATHS-LG + GapCloser
Scaffold	Total length (bp)	157,408,176	146,937,509	157,284,427
	Number	1,538	16,684	1,536
	N50 length (bp)	806,518	187,271	805,959
	Longest scaffold (bp)	7,037,412	2,341,668	7,038,285
	Gap size (bp)	18,606,682	26,830,789	8,276,177
Contig	Total length (bp)	138,806,556	120,116,579	149,009,524
	Number	14,076	37,240	7,472
	N50 length (bp)	25,949	4,905	48,580
	Longest contig (bp)	417,030	70,238	788,128
BUSCO	Total complete	87.80%	77.20%	89.80%
	Single copy complete	86.20%	75.90%	88.00%
	Duplicated complete	1.60%	1.30%	1.80%
	Fragmented	3.50%	7.90%	2.20%
	Missing	8.70%	14.90%	8.00%
	Total number	1440	1440	1440

The ALLPATHS-LG assembly was further refined by filling gaps and removing scaffolds from contaminant species. Gaps were reduced in the assembly from 13,729, accounting for 18,606,682 bp or 11.8% of the assembly, to 5068, accounting for 8,276,177 bp or 5.3% of the new gap-closed assembly. Two scaffolds in the gap-closed assembly had matches (>95% identity and >50% coverage) to spider mite (*Tetranychus urticae*) ribosomal DNA and were removed, generating a final genomic assembly of 157,284,427 bp with an N50 scaffold size of 805,959 bp (Table 1).

To assess the completeness and accuracy of the final assembly, the presence of conserved plant orthologs and the numbers of nucleotide variants were determined. A total of 92.0% (89.8% complete and 2.2% fragmented) of BUSCO orthologs were present in the assembly (Table 1), indicating a near complete representation of the genic space. After marking PCR duplicates, 94.6% of the reads mapped to the final genomic assembly; of the reads that mapped, 95.0% were properly paired. Using these alignments, a total of 1,270,680 initial variants (SNPs and InDels) were identified and reduced to 593,265 after filtering with the GATK VariantFiltration tool (McKenna *et al.* 2010), and separately by allele frequency, with 364,465 variants being heterozygous sites and 228,800 representing assembly errors. The heterozygous and assembly error variants account for 0.23 and 0.15% of the assembly, respectively, indicative of a relatively error-free and slightly heterozygous genome with one heterozygous variant per 432 bp.

### Quality assessment of de novo-assembled transcriptomes and genome annotation

To determine if any transcripts represented potential contaminants, such as microbial or insect RNA commonly present in greenhouse-grown plants, *de novo*-assembled transcriptomes from each RNA-seq library (Table S4 in File S1) were aligned to the genome and searched against the NCBI nr database. Between 65.7 and 71.6% of unaligned transcripts contained a match to the database, with 80.7–98.7% of these containing a best match to a species in the Viridiplantae kingdom. Libraries from the sepal/petal and gynostegium tissues (SRR6078591 and SRR6078593, respectively) contained a higher percentage of best matches to non-Viridiplantae species at 14.5 and 19.3% (Table S5 in File S1), respectively, with thrips (*Frankliniella occidentalis*), an insect typically associated with floral structures, the most prevalent species. After removing the non-Viridiplantae transcripts, between 93.1 and 94.2% of all transcripts for each library aligned to the genome, with 90.1–91.5% aligning uniquely (Table S5 in File S1). Excluding SRR6078591 and SRR6078593, which had the most contamination with nonplant sequences, between 93.2 and 94.3% of the RNA-seq reads aligned to the genome assembly (Table S5 in File S1), consistent with the percentage of *de novo*-generated Trinity transcripts that aligned to the genome after removal of contamination and indicating a robust representation of the genic space.

After confirming the quality of the RNA-seq data, genome annotation was performed. A total of 117,995 repetitive elements, representing 28.3% of the genome assembly, were identified (Table S6 in File S1). The final gene model working set consisted of 19,536 loci and 22,218 gene models, with 20,832 gene models (18,197 loci) identified as high confidence. A total of 18,436 high-confidence gene models were assigned a putative function, 1264 were annotated as conserved hypothetical, and 1132 were annotated as hypothetical.

### Homologous and syntenic relationships in the Apocynaceae family

**Figure 3:**
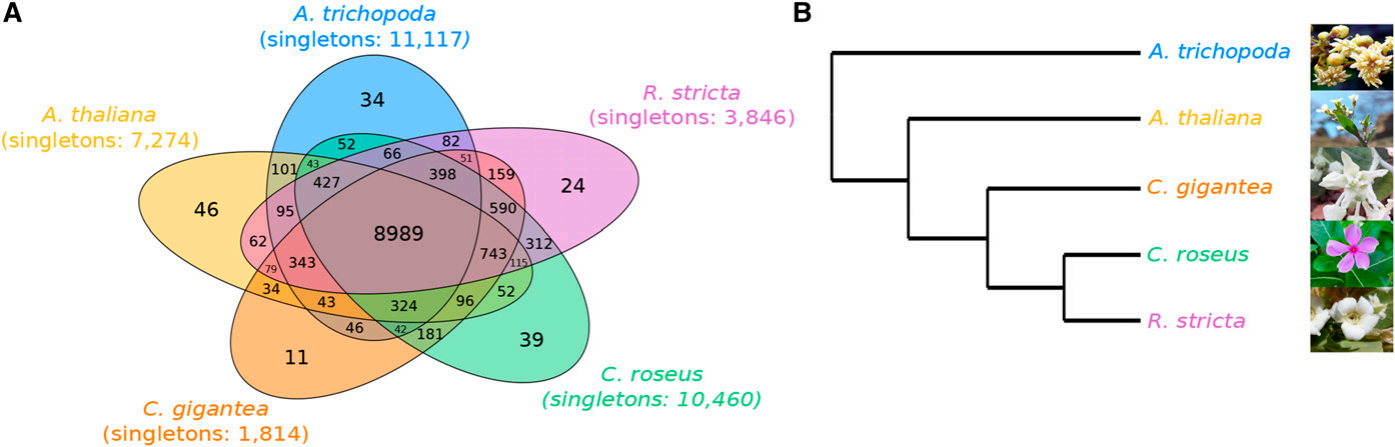
Homologous and syntenic relationships among the Apocynaceae family. (A) Venn diagram showing the number of orthologous and paralogous groups shared among the species. The number of singletons per species is also provided. (B) Rooted cladogram for the species generated in the OrthoFinder (Emms and Kelly 2015) output.

The Apocynaceae family is diverse, containing five subfamilies and 400 genera that produce diversified specialized metabolites (http://www.mobot.org/MOBOT/Research/APweb/welcome.html). Despite the large number of species in the family, only two have an annotated genome assembly, *C. roseus* and *R. stricta* (Kellner *et al.* 2015; Sabir *et al.* 2016), both of which are in the Rauvolfioideae subfamily. A total of 13,679 orthologous groups were identified from comparisons between *A. trichopoda*, *A. thaliana*, *C. roseus*, *R. stricta*, and *C. gigantea* (Table S3 in File S1), of which 8989 groups contained all five species (Figure 3). *C. gigantea* had few singleton genes, with 90.0% of its genes, the most of any species, in 12,118 orthologous groups and 11 paralogous groups; *C. gigantea*-specific genes included disease-resistance genes, F-box family proteins, and hypothetical genes suggestive of genes rapidly evolving (Table S7 in File S1). We identified 590 Apocynaceae-specific orthologous groups containing only genes from *C. gigantea*, *C. roseus*, and *R. stricta*, whereas 312 orthologous groups contained only *C. roseus* and *R. stricta*, almost double the number shared between *C. gigantea* and *C. rosesus* or *C. gigantea* and *R. stricta*, highlighting the evolutionary relationships between the species, as *C. roseus* and *R. stricta* are in the Rauvolfioideae subfamily while *C. gigantea* is in the Asclepiadoideae subfamily (Chan *et al.* 2016; Sabir *et al.* 2016). The rooted species tree from OrthoFinder (Emms and Kelly 2015) (Figure 3B), constructed using the median distances for the genes in orthologous groups, supports this relationship.

Syntenic relationships among the species in the Apocynaceae family were determined to further examine the evolutionary relationships. A total of 1836 collinear blocks were present in a comparison of all three species with each species containing a similar number of collinear genes (∼12,500–13,000) (Table S8 in File S1). In pairwise comparisons, *C. roseus* and *C. gigantea* each had 549 and 482 more collinear genes, respectively when paired together compared to when either was paired with *R. stricta*; however, *R. stricta* had virtually no difference in the number of collinear genes between its pairing with *C. gigantea* and *C. roseus* (Table S8 in File S1). The relatively small difference in number of collinear genes between the three species, despite there being almost twice as many orthologous groups between *C. roseus* and *R. stricta* compared to *C. gigantea*, could be due to a number of reasons, including: a dispersed distribution of orthologous genes in the genome thereby not meeting our threshold for a collinear block, differences in gene annotation methods among the three genomes, and the collective fragmentation of the assemblies from the three species. Regardless, the expected evolutionary relationships were reflected in the orthologous groupings and suggest the emergence or loss of the cardenolide metabolites subsequent to the divergence of the Apocynaceae subfamilies, as *C. roseus* and *R. stricta* do not produce cardenolides.

### Future uses for the C. gigantea genome assembly and annotation

Cardenolides, specialized compounds produced by *C. gigantea*, are known to contain anticancer and antimalarial properties (Chan *et al.* 2016). Yet, while much is known about the compounds themselves, knowledge of the biosynthetic pathways involved is limited (Bauer *et al.* 2010; Munkert *et al.* 2014; Pandey *et al.* 2016). Furthermore, to the best of our knowledge, no nuclear genome assembly has been published for any cardenolide-producing species in the Asterid clade. The *C. gigantea de novo* genome assembly, annotation, and RNA-seq expression data provide a valuable new resource for the further study of the biosynthesis of cardenolides with applications in the pharmaceutical industry.

**Figure 4:**
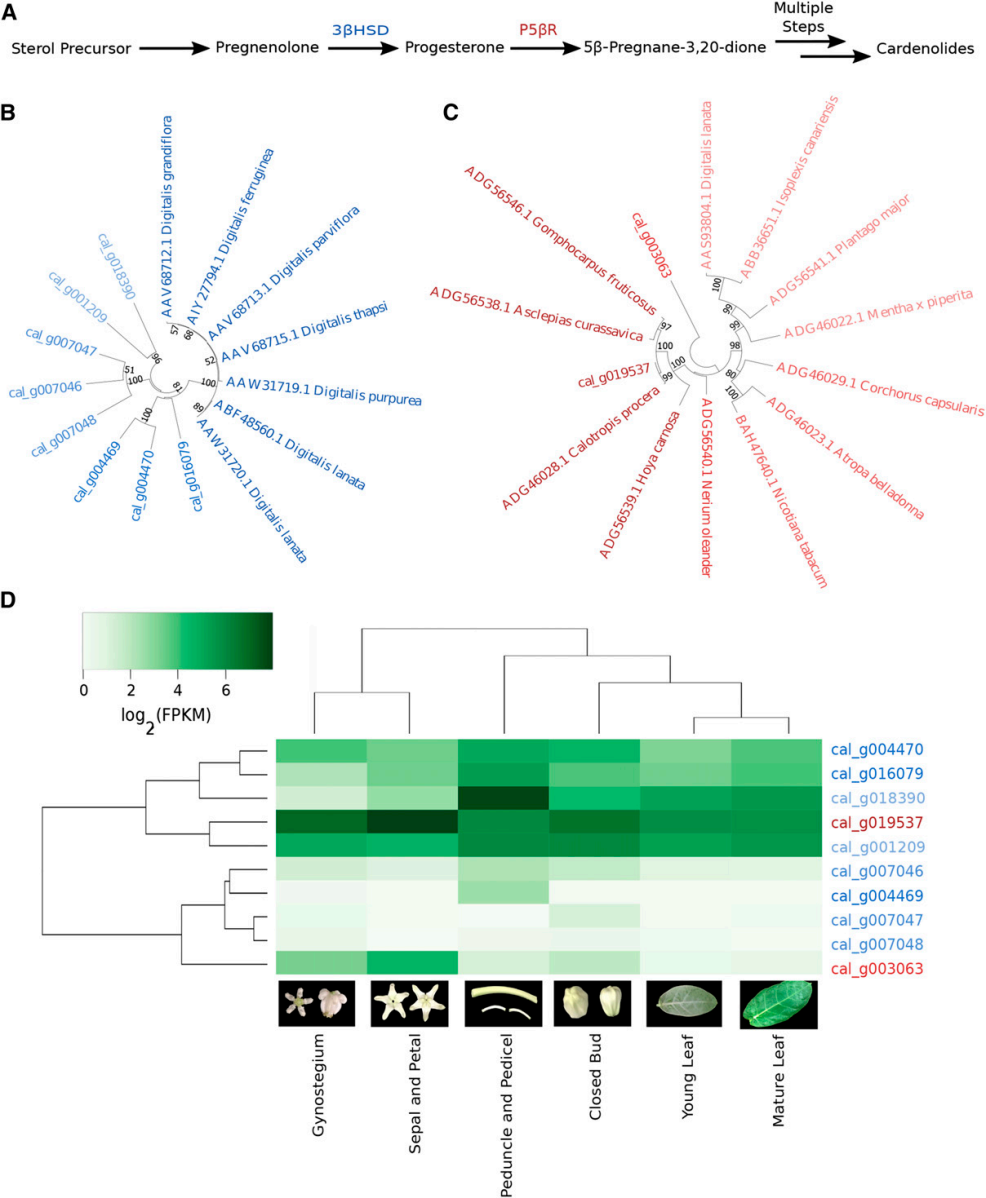
Identification of putative *C. gigantea* genes involved in the cardenolide biosynthetic pathway. (A). Simplified schematic of the cardenolide biosynthetic pathway. (B and C) are neighbor-joining gene trees for 3β-hydroxysteroid dehydrogenase (3βHSD) and progesterone 5β-reductase (P5βR), respectively. Taxa for each tree are the *C. gigantea* candidates and functionally characterized proteins, for which the taxa labels include the GenBank identifier and species name; taxa are shaded according to their distance from the functionally characterized genes, with darker shades indicating smaller distances. Values on nodes indicate bootstrap support from 1000 bootstrap replicates. (D) Heat map of log2-transformed gene expression values (FPKM: fragments per kb exon model per million mapped reads) of candidate *C. gigantea* cardenolide biosynthesis genes. Cladograms were generated from conducting hierarchical clustering on the genes and samples. Blue and red colored genes are 3βHSD and P5βR candidates, respectively.

With the use of this resource, both evolutionary and coexpression relationships can be utilized to elucidate genes involved in the cardenolide biosynthetic pathway. As demonstrated here, comparative genomic analyses between *C. gigantea* and other species in the Apocynaceae family can elucidate evolutionary events that led to the emergence of their corresponding specialized secondary metabolites. For example, identification of orthologous genes and syntenic blocks can facilitate the elucidation of genes of interest in the cardenolide biosynthetic pathway as they may be enriched or unique to *C. gigantea*, or physically clustered in the genome as shown in other species (Denoeud *et al.* 2014; Nützmann *et al.* 2016; Sabir *et al.* 2016). Another method used to identify candidate specialized metabolic pathway genes is coexpression (Itkin *et al.* 2013; Kellner *et al.* 2015), which the developmental *C. gigantea* RNA-seq data sets can facilitate. Two enzymes known to catalyze key parts of the cardenolide biosynthetic pathway in other cardenolide-producing species (Figure 4A) were examined in *C. gigantea*; eight and two *C. gigantea* genes had sequence similarity to functionally characterized 3βHSD (Herl *et al.* 2007) and P5βR (Bauer *et al.* 2010) proteins, respectively (Table S9 in File S1). Neighbor-joining gene trees with the *C*. *gigantea* candidates, and functionally characterized 3βHSD and P5βR genes, indicate that three genes (cal_g004469, cal_g004470, and cal_g016079), classified as paralogous by OrthoFinder, are most closely related to *Digitalis* 3βHSD proteins (Figure 4, B and C). Of the two *C. gigantea* P5βR paralogous genes, cal_g019537 was closely related to the *Calotropis procera* P5βR protein. Hierarchical clustering of normalized expression values for putative *C. gigantea* 3βHSD and P5βR genes indicates that the putative 3βHSD gene cal_g001209 and the putative P5βR gene cal_g019537 are coordinately expressed (Figure 4D), and are strong candidates for functional validation of the cardenolide biosynthetic pathway in *C. gigantea*. This case study demonstrates the capability of the *C. gigantea de novo* genome assembly, annotation, and RNA-seq expression data in determining putative genes involved in the cardenolide biosynthetic pathway.

## Supplementary Material

Supplemental material is available online at www.g3journal.org/lookup/suppl/doi:10.1534/g3.117.300331/-/DC1.
