# Supplementary material for: Genome Assembly and Annotation of the Medicinal Plant Calotropis gigantea, a Producer of Anticancer and Antimalarial Cardenolides
Source: G3 (Bethesda). 2017 Dec 12;8(2):385–91. doi: 10.1534/g3.117.300331 (PMC5919723; doi:10.1534/g3.117.300331)
Supplement: Supplementary file 1 [file 385FileS1.docx]

**Table S1: Genomic Libraries Used in the Genome Assembly**

|  |  |  |  | **Use** | |
| --- | --- | --- | --- | --- | --- |
|  | **SRA Accession** | **Fragment Size (bp)** | **No. of cleaned Reads** | **ALLPATHS-LG Assembly** | **Platanus Assembly** |
| **Fragment** | SRR6078579 | 168 | 102,328,102 | ALLPATHS-LG | Platanus |
|  | SRR6078578 | 168 | 101,964,888 | ALLPATHS-LG | Platanus |
|  | SRR6078581 | 291 | 27,663,060 | GapCloser | Platanus |
|  | SRR6078580 | 291 | 25,014,954 | GapCloser | Platanus |
|  | SRR6078583 | 519 | 10,285,242 | GapCloser | Platanus |
|  | SRR6078582 | 519 | 11,508,512 | GapCloser | Platanus |
| **Mate Pair** | SRR6078585 | 2,664 | 9,220,004 | ALLPATHS-LG | Platanus |
|  | SRR6078584 | 4,739 | 11,943,572 | ALLPATHS-LG | Platanus |
|  | SRR6078587 | 7,678 | 8,868,446 | ALLPATHS-LG | Platanus |
|  | SRR6078586 | 8,793 | 4,200,226 | ALLPATHS-LG | Platanus |

**Table S2: RNA Libraries Used in This Study.**

| **SRA Accession** | **Tissue** | **No. of Cleaned Reads** |
| --- | --- | --- |
| SRR6078591 | Sepal and Petal | 39,594,585 |
| SRR6078590 | Closed Bud | 38,775,388 |
| SRR6078589 | Young Leaf | 23,775,669 |
| SRR6078588 | Peduncle and Pedicel | 30,591,663 |
| SRR6078593 | Gynostegium | 27,286,590 |
| SRR6078592 | Mature Leaf | 34,563,990 |

**Table S3: Species Used in Homologus and Syntenic Analyses**

| **Species** | **Genome Assembly Size (bp)** | **Scaffold N50 Size (bp)** | **Annotation Version** | **No. of Genes** | **Reference** |
| --- | --- | --- | --- | --- | --- |
| *Amborella trichopoda* | 706,332,648 | 4,927,027 | v1 | 26,846 | (Amborella Genome Project 2013) |
| *Arabidopsis thaliana* | 119,667,750 | 23,459,830 | TAIR10 | 27,416 | (Lamesch *et al.* 2012) |
| *Catharanthus roseus* | 541,127,784 | 2,579,454 | v2 | 34,363 | Unpublished |
| *Rhayza stricta* | 274,354,041 | 5,553,863 | v1 | 21,164 | (Sabir *et al.* 2016) |

|  | **SRR6078591** | **SRR6078590** | **SRR6078589** | **SRR6078588** | **SRR6078593** | **SRR6078592** |
| --- | --- | --- | --- | --- | --- | --- |
| **Tissue** | Sepal and Petal | Closed Bud | Young Leaf | Peduncle and Pedicel | Gynostegium | Mature Leaf |
| **Total Length (bp)** | 16,934,918 | 15,727,281 | 14,703,954 | 15,531,070 | 14,889,374 | 16,370,369 |
| **No. of ‘genes’** | 14,860 | 15,562 | 14,473 | 14,571 | 13,948 | 15,750 |
| **No. of transcripts** | 16,173 | 15,946 | 14,750 | 15,523 | 14,864 | 16,215 |
| **Transcript N50 (bp)** | 1,302 | 1,099 | 1,105 | 1,180 | 1,180 | 1,142 |
| **Mean Transcript Length (bp)** | 1,139.6 | 1,010.6 | 1,015.9 | 1,065.9 | 1,067.5 | 1,039.4 |

**Table S4: *de novo* Trinity Assembled Transcriptome Metrics**

Transcriptome metrics based on longest isoform for each gene only after filtering for transcripts larger than or equal to 500 bp.

|  |  | | **SRR6078591** | | **SRR6078590** | **SRR6078589** | **SRR6078588** | **SRR6078593** | **SRR6078592** |
| --- | --- | --- | --- | --- | --- | --- | --- | --- | --- |
|  | **Tissue** | | Sepal and Petal | | Closed Bud | Young Leaf | Peduncle and Pedicel | Gynostegium | Mature Leaf |
| **BLASTN*^a^*** | | **Non-Viridiplantae** | | 14.46% | 1.80% | 4.74% | 2.04% | 19.27% | 1.27% |
| **GMAP*^b^*** | | **Unique** | | 89.88% | 91.33% | 92.04% | 90.52% | 90.10% | 91.48% |
|  | | **Multiple** | | 0.49% | 0.48% | 0.46% | 0.39% | 0.45% | 0.40% |
|  | | **Cross-Scaffold** | | 2.88% | 2.09% | 1.70% | 2.69% | 2.51% | 2.02% |
|  | | **None** | | 7.48% | 6.18% | 6.01% | 6.49% | 7.94% | 6.15% |
| **TopHat2*^c^*** | | **Read Alignment** | | 91.20% | 93.20% | 94.20% | 93.90% | 91.00% | 94.30% |

**Table S5: RNA-sequencing read contaminant and alignment metrics.**

*^a^*Total percentage of non-mapping transcripts with BLASTN best matches to species in the non-Viridiplantae kingdom.

***^b^***GMAP alignment metrics are based on a 95% coverage and identity cutoff after removing unaligning non-Viridiplantae transcripts.

*^c^*TopHat2 alignments for all reads, with contaminants present in SRR6078591 and SRR6078593 libraries leading to a lower percentage of read alignment.

**Table S6: Repetitive Content in the *Calotropis gigantea* Genome Assembly**

|  | **No. Elements** | **Length Occupied (bp)** |
| --- | --- | --- |
| **SINEs** | 41 | 2,915 |
| **LINEs** | 2,213 | 2,385,297 |
| **LTR Elements** | 35,422 | 24,701,874 |
| **DNA Elements** | 31,893 | 3,455,204 |
| **Unclassified** | 48,426 | 14,099,267 |
| **Total** | 117,995 | 44,644,557 |

**Table S7: *C. gigantea* specific paralogous groups**

| **Paralogous Group** | **Gene** | **Functional annotation** |
| --- | --- | --- |
| OG0000464 | cal_g008172.t1 | Ribonuclease H-like superfamily protein |
|  | cal_g010091.t1 | hypothetical protein |
|  | cal_g011398.t1 | hypothetical protein |
|  | cal_g011552.t1 | Glycine rich protein family domain containing protein |
|  | cal_g012287.t1 | Ribonuclease H-like superfamily protein |
|  | cal_g015205.t1 | hypothetical protein |
|  | cal_g015225.t1 | Ribonuclease H-like superfamily protein |
|  | cal_g015436.t1 | Reverse transcriptase-like domain containing protein |
|  | cal_g015490.t1 | hypothetical protein |
|  | cal_g016588.t1 | Reverse transcriptase-like domain containing protein |
|  | cal_g017228.t1 | Ribonuclease H-like superfamily protein |
|  | cal_g018672.t1 | hypothetical protein |
|  | cal_g018743.t1 | hypothetical protein |
|  | cal_g019010.t1 | hypothetical protein |
|  | cal_g019342.t1 | Polynucleotidyl transferase, ribonuclease H-like superfamily protein |
| OG0002847 | cal_g011920.t1 | Disease resistance protein (CC-NBS-LRR class) family |
|  | cal_g012934.t1 | Disease resistance protein (CC-NBS-LRR class) family |
|  | cal_g012935.t1 | Disease resistance protein (CC-NBS-LRR class) family |
|  | cal_g017797.t1 | Disease resistance protein (CC-NBS-LRR class) family |
|  | cal_g017799.t1 | LRR and NB-ARC domains-containing disease resistance protein |
|  | cal_g017800.t1 | Disease resistance protein (CC-NBS-LRR class) family |
|  | cal_g017801.t1 | Disease resistance protein (CC-NBS-LRR class) family |
| OG0003754 | cal_g006816.t1 | S-adenosyl-L-methionine-dependent methyltransferases superfamily protein |
|  | cal_g006817.t1 | S-adenosyl-L-methionine-dependent methyltransferases superfamily protein |
|  | cal_g006870.t1 | S-adenosyl-L-methionine-dependent methyltransferases superfamily protein |
|  | cal_g006871.t1 | jasmonic acid carboxyl methyltransferase |
|  | cal_g006872.t1 | S-adenosyl-L-methionine-dependent methyltransferases superfamily protein |
|  | cal_g014927.t1 | jasmonic acid carboxyl methyltransferase |
| OG0005599 | cal_g010656.t1 | HXXXD-type acyl-transferase family protein |
|  | cal_g011835.t1 | HXXXD-type acyl-transferase family protein |
|  | cal_g011836.t1 | HXXXD-type acyl-transferase family protein |
|  | cal_g015379.t1 | HXXXD-type acyl-transferase family protein |
|  | cal_g015541.t1 | HXXXD-type acyl-transferase family protein |
| OG0005604 | cal_g011973.t1 | RING/U-box superfamily protein |
|  | cal_g011976.t1 | RING/U-box superfamily protein |
|  | cal_g013493.t1 | hypothetical protein |
|  | cal_g016425.t1 | COP1-interacting protein |
|  | cal_g016426.t1 | RING/U-box superfamily protein |
| OG0005605 | cal_g015254.t1 | hypothetical protein |
|  | cal_g017687.t1 | hypothetical protein |
|  | cal_g019066.t1 | hypothetical protein |
|  | cal_g019067.t1 | hypothetical protein |
|  | cal_g019068.t1 | hypothetical protein |
| OG0010421 | cal_g013175.t1 | hypothetical protein |
|  | cal_g013764.t1 | extensin proline-rich |
|  | cal_g013765.t1 | hypothetical protein |
|  | cal_g016563.t1 | ECA1 gametogenesis related family protein |
| OG0012148 | cal_g008960.t1 | Ribonuclease H-like superfamily protein |
|  | cal_g012279.t1 | Reverse transcriptase-like domain containing protein |
|  | cal_g014860.t1 | Polynucleotidyl transferase, ribonuclease H-like superfamily protein |
| OG0012311 | cal_g013424.t1 | hypothetical protein |
|  | cal_g014988.t1 | hypothetical protein |
|  | cal_g017733.t1 | hypothetical protein |
| OG0012351 | cal_g012987.t1 | F-box family protein |
|  | cal_g012988.t1 | F-box family protein |
|  | cal_g012989.t1 | F-box family protein |
| OG0012388 | cal_g014825.t1 | Ribonuclease H-like superfamily protein |
|  | cal_g015158.t1 | hypothetical protein |
|  | cal_g015176.t1 | hypothetical protein |

Functional annotation for *C. gigantea* genes found in the paralogous groups.

**Table S8: Syntenic Relationships Among the Apocynaceae Family**

|  | ***C. gigantea* vs. *C. roseus*** | ***C. gigantea* vs. *R. stricta*** | ***C. roseus* vs. *R. stricta*** | ***C. gigantea* vs. *C. roesus* vs. *R. stricta*** |
| --- | --- | --- | --- | --- |
| ***C. gigantea*** | 11,706 | 11,224 | N/A | 13,270 |
| ***C. roseus*** | 11,491 | N/A | 10,942 | 13,176 |
| ***R. stricta*** | N/A | 10,976 | 10,956 | 12,473 |

Number of genes per species in collinear pairs for each compairson.

|  | ***C. gigantea* Gene** | **Subject Match (GenBank ID)** | **Query Coverage** | **Subject Coverage** | **Percent Identity** | **E-value** |
| --- | --- | --- | --- | --- | --- | --- |
| **3βHSD** | cal_g007048.t1 | *D. lanata* (AAW31720.1) | 97% | 97% | 50.19% | 1.16E-79 |
|  | cal_g007046.t1 | *D. lanata* (AAW31720.1) | 88% | 97% | 50.39% | 4.49E-90 |
|  | cal_g018390.t1 | *D. lanata* (AAW31720.1) | 91% | 97% | 50.397 | 1.59E-85 |
|  | cal_g001209.t2 | *D. ferruginea*  (AIY27794.1) | 91% | 98% | 50.575 | 7.26E-82 |
|  | cal_g007047.t1 | *D. lanata* (AAW31720.1) | 96% | 97% | 54.76% | 6.69E-94 |
|  | cal_g004470.t1 | *D. lanata* (AAW31720.1) | 95% | 99% | 56.42% | 3.05E-103 |
|  | cal_g004469.t1 | *D. parviflora*  (AAV68713.1) | 96% | 98% | 55.34% | 2.35E-99 |
|  | cal_g016079.t1 | *D. lanata* (AAW31720.1) | 97% | 99% | 60.54% | 3.25E-108 |
| **P5βR** | cal_g019537.t1 | *C. procera*  (ADG46028.1) | 100% | 100% | 99.49% | < 1E-180 |
|  | cal_g003063.t1 | *N. oleander*  (ADG56540.1) | 100% | 100% | 72.56% | < 1E-180 |

**Table S9: *Calotropis gigantea* BLAST Matches to 3βHSD and P5βR** **proteins**

Metrics on *C. gigantea* protein matches to 3β-hydroxysteroid dehydrogenase (3βHSD) and progesterone 5β-reductase (P5βR) proteins.
